# Naturalist's Monthly Report

**Published:** 1812-03

**Authors:** 


### NATURALIST'S MONTHLY REPORT.

### JANUARY.

REVIVING WINTER MONTH.

Now joyless rains obscure,
Drive through the mingling skies with vapour foul;
Dash on the mountain's brow, and shake the woods.

FROM the 1st to the 4th of this month the wind was either westerly or south-west; on the 5th and 6th, variable; from the 7th to the 10th, north-east; on the 11th, northerly; on the 12th, east; from from the 13th to the 16th, northerly; on the 17th, east; on the 18th, variable; from the 19th to the 22d, north west; on the 23d and 24th, east and north-north-east; and from the 25th to the end of the month, westerly and south-west.

There were fresh gales on the 4th, 7th, 19th, 20th, 21st, 25th, 27th, and 30th; strong gales on the 2d, 3d, 5th, 8th, 23d, 28th, and 31st; and hard gales on the 29th.

The rainy days were the 2d, 3d, 4th, 5th, 10th, 29th, 30th, and 31st.

January 1st. The redbreast sings. The weather still continues extremely mild. Moles throw up hillocks; and several species of beetles are to be seen flying and crawling about.

January 5th. We had some snow, but it dissolved almost immediately after it fell upon the ground.

January 7th. The large green woodpecker *(picus viridis)* is heard.

January 8th. The mail-coach was detained for a considerable length of time upon the road, by the snow that had fallen; and the higher parts of the New Forest, as well as the hills of Dorsetshire, appear covered with snow; but here, in the immediate neighbourhood of the sea, we have had none since Sunday last, the 5th instant.

January 9th. The flowers of the spurge laurel *(Daphne laureola)* are formed, and the petals appear nearly ready to expand. This is the first real indication of returning spring that I have hitherto seen.

In the evening of this day there was a considerable fall of snow; but it only continued on the ground till the following morning.

January 12th. Small birds, and particularly the titmice, tear off moss from the trees, in pursuit of insects which lie concealed beneath. Their agility in this operation is very pleasing.

January 16th. Slugs and worms come out of their holes in the night. The skylark and blackbird are heard.

January 17th. Beans and pease that were planted in the beginning of November are now three or four inches above the ground.

January 18th. Sea-gulls frequent the interior parts of the country.

January 19th. The first leaves of navel-wort *(cotyledon umbilicus)*, and wake-robin or cuckoo-pint *(arum maculatum)*, appear.

January 21st. There was a sharp frost in the night; and we also had frosty weather on the 23d and 24th, with the easterly winds which prevailed on those days.

January 26th. The winter aconite *(helleborus hyemalis)* is in flower. The flowers of the snow-drop and leaves of the crocus appear above the ground.

The chaffinch and hedge-sparrow sing.

January 28th. A dormouse was this day brought to me. It had been found, in a torpid state, in its winter's nest, but had revived from its torpidity before I received it.

January 30th. The beautiful crimson styles of the female flowers of the hazel, are now fully expanded; and the catkins begin to open.

January 31st. The mezereon and hepatica are in flower.

*Hampshire.*

